# The impact of environmental factors on traffic accidents in Iran

**DOI:** 10.5249/jivr.v6i2.318

**Published:** 2014-07

**Authors:** Kamran B. Lankarani, Seyed Taghi Heydari, Mohammad Reza Aghabeigi, Ghasem Moafian, Amin Hoseinzadeh, Mehrdad Vossoughi

**Affiliations:** ^*a*^Health Policy Research Center, School of Medicine, Shiraz University of Medical Sciences, Shiraz, Iran.; ^*b*^Jahrom University of Medical Sciences, Jahrom, Iran.; ^*c*^Department of Dental Public Health, School of Dentistry, Shiraz University of Medical Sciences, Shiraz, Iran.

**Keywords:** Road traffic crashes, Injury, Mortality, Environmental factors, Iran

## Abstract

**Background:**

Road traffic crashes are the third highest cause of mortality in Iran. The aim of this study was to determine the impact of roadway environmental factors on traffic crash.

**Methods:**

This cross-sectional study was conducted in Iran between March 21, 2010 and December 30, 2010. The data on road traffic crashes were obtained from the Traffic Police Department records. These records were classified to control for the main confounders related to the type of crash and roadway environmental factors. Roadway environmental factors included crash scene light, weather, place of accident, the defects and geometrics of roadway and road surface.

**Results:**

The study included 542,863 traffic crashes. The proportions of road traffic crash which led to injury were 24.44% at sunrise and 27.16% at sunset compared with 5.43% and 1.43% deaths at sunrise and sunset respectively. In regard to day time accidents, the proportions were 20.50% injuries and 0.55% deaths. The statistical analysis of the results showed that the ratio of injuries and deaths were significantly higher at sunrise and sunset than those occurring during daytime (P less than 0.001). The highest rate of death (5.07%) was due to dusty weather compared to 5.07% for other weather conditions (P less than 0.001). The highest mortality rate (3.45%) occurred on oily surfaces (P less than 0.001). The defective traffic signs were responsible for 30,046 injuries and 5.58% deaths, and road narrowing accounted for 22,775 injuries and, 4.23% deaths which indicated that the roadway defects inflict most frequent injuries and deaths. The lowest (0.74 %) and highest (3.09%) proportion of traffic crash- related deaths were due to flat straight and winding uphill/downhill roads respectively (P less than 0.001).

**Conclusions:**

Sunrise, sunset, dusty weather, oily road surfaces and winding uphill/downhill road were hazardous environmental factors. This study provides an insight into the potential impacts of environmental factors on road traffic accidents and underlines the implementation of appropriate preventive measures.

## Introduction

According to the recently published World Health Organization (WHO) global status report on road safety, annually road traffic crashes (RTCs) injure about 50 millions and kill as many as 1.2 million people worldwide.^1^ Exposure to RTCs is a serious public health challenge in Iran^2-4^ because of a variety of reasons. These include the tendency of young population to adventurous driving, low gas price, the use of private vehicles rather than public transport, and non-standard safety designs.^4^ Also, with respect to the oil and gas economy, the growth of Iran’s automotive industry led to a significant increase in the number of registered vehicles to 17 millions in 2008.^4^ The WHO report shows that 22,918 road traffic fatalities occurred in Iran during 2007-2008.^5^ According to the estimate reported in 2005,^6^ the mortality due to traffic crashes in Iran rated highest among all countries of the world.

The effect of environment on several health issues has been an interesting topic for researchers for a long time. For instance, meteorological factors are known to modulate human health status.^6^ In the field of RTCs, a growing body of studies has reviewed the relationship between environmental factors and RTCs.^7-10^ Some reports analyzed pedestrian volume, traffic volume, roadway geometrics, weather and other seasonal effects as environmental factors.^7-9^ Others discussed the effect of light condition during day and night on RTCs. 

Several studies have focused on different aspects of RTCs in Iran during recent years.^11-14^ However; there is no published report on the impact of environmental factors on RTCs. The aim of the present study was to determine the association between environmental factors and RTCs in Iran so as to provide an insight into potential effect of environmental factors on RTCs and to improve preventive measures. 

## Methods

This cross-sectional study was conducted in Iran from March 21, 2010 to December 30, 2010. The most recent and reliable RTC data were obtained from the center for information and communication technology (ICT) of the Traffic Police Department. 

These included 542,863 RTC records which were classified to control for the main confounders associated with crash type involving death in the scene of crash, injurious and benign traffic accidents and roadway-associated environmental factors. Roadway environmental factors involve light in the scene of crash (daytime, nighttime, sunrise and sunset), weather conditions such as clear, foggy, snowy, rainy, stormy, cloudy, and dusty, place of crash (motorway, road shoulder, middle lane, roadside, outside of road limit and unknown), roadway conditions ( good ,damaged , defective traffic signs, road narrowing, bumps, unstable shoulders or lack of shoulders, absence of standard road guard, partial road collapse, defective pavements, acute angle, non-standard grading, defective lighting, slippery and unknown), roadway geometrics (straight and flat, straight with uphill/downhill orientations, winding and flat road, winding and uphill/downhill road) and roadway surface (dry, wet, freezing and snowy, sandy, muddy, and oily). The characteristics of each confounder were then determined to analyze the association between roadway environmental factors and RTCs.

All data were analyzed using SPSS statistical software, version 11.5. The results are reported as descriptive indices such as frequency (percentage). Comparisons between the type of crash and other variables were done using chi-squared test. Multinomial logistic regression was used to assess the odds of type of crash (injury and death in scene) relative to the reference group of non-injured vehicular crash. P value less than 0.05 was considered significant.

## Results

The crash and environment-related information of the total RTCs are given in Table 1. Table 2 and 3 represent a compilation of the type of crash according to a number of environment and road- related factors. Of all entities demonstrated in foregoing Tables, scene light, weather condition, place of crash, roadway defect, roadway geometrics and roadway surface were significantly associated with type of crash. 

**Table 1:** Crashes and environmental related data of the total RTCs during the study period.

Variable		Frequency	Percentage
	Non-injured vehicular crash	427643	78.78
Type of crash	Injury	111832	20.60
	Death in the scene	3388	0.62
	Total	542863	100.0
	Day	393310	72.45
	Night	122957	22.65
Scene light	Sunrise	1252	0.23
	Sunset	5660	1.04
	Unknown	19684	3.63
	Clear	471759	86.90
	Foggy	1508	0.28
	Snowy	1761	0.32
Weather	Rainy	22442	4.13
	Stormy	148	0.03
	Cloudy	26198	4.83
	Dusty	217	0.04
	Unknown	18830	3.47
	Straight and flat	346168	63.77
	Straight with uphill/downhill	12827	2.36
Roadway geometrics	Winding road and flat	16002	2.95
	Winding road with uphill/downhill	4862	0.90
	Unknown	163004	30.03
	Dry	492757	90.77
	Wet	25989	4.79
	Freezing and snowy	1280	0.24
Roadway surface	Sandy	1118	0.21
	Muddy	85	0.02
	Oily	29	0.01
	Unknown	21604	3.98
	No defect	204565	37.98
	Damaged or defective traffic signs	30046	5.58
	Road narrowing	22775	4.23
	Bumps	1718	0.32
	Unstable shoulders or nonexistent shoulders	943	0.18
	Lack of standard road guard	1384	0.26
Roadway defect	Partial road collapse	201	0.04
	Defective pavements	376	0.07
	Acute angle	695	0.13
	Non-standard grading	360	0.07
	Defective lighting	968	0.18
	Slippery road	934	0.17
	Unknown	273623	50.80

**Table 2:** Evaluation of the type of crash with respect to a number of environments related factors.

Environment related factors		Type of crash			P value
		Non-injured vehicular crash (%)	Injury(%)	Death in scene (%)	
	Day	310537 (78.95)	80625 (20.50)	2148 (0.55)	
	Night	92504 (75.23)	29362 (23.88)	1091 (0.89)	
Scene light	Sun rise	878 (70.13)	306 (24.44)	68 (5.43)	<0.001
	Sun set	4042 (71.41)	1537 (27.16)	81 (1.43)	
	Unknown	19682 (99.99)	2 (0.01)	0 (0.00)	
	Clear	365249 (77.42)	103542 (21.95)	2968 (0.63)	
	Foggy	1216 (80.64)	273 (18.10)	19 (1.26)	
	Snowy	1580 (89.72)	165 (9.37)	16 (0.91)	
Weather	Rainy	19385 (86.38)	2908 (12.96)	149 (0.66)	<0.001
	Storm	111(75.00)	35 (23.65)	2 (1.35)	
	Cloudy	21127 (80.64)	4848 (18.51)	223(.85)	
	Dust	146 (67.28)	60 (27.65)	11(5.07)	
	Unknown	18828 (99.99)	1(0.01)	0 (0.00)	

**Table 3:** Evaluation of the type of crash with respect to a number of road related factors.

Road related factors		Type of crash			P value
		Non-injured vehicular crash (%)	Injury(%)	Death in scene (%)	
	Motorway	268951(77.13)	77235(22.15)	2524(0.72)	
	Road shoulder	2826(71.49)	993(25.12)	134(3.39)	
Place of crash	The middle lane	1742(80.31)	388(17.89)	39(1.80)	<0.001
	Roadside	2953(60.95)	1651(34.08)	241(4.97)	
	Outside of road limit	842(72.03)	283(24.21)	44(3.76)	
	Unknown	146618(82.49)	30751(17.30)	373(0.21)	
	No defect	160674(78.54)	42121(20.59)	1770(0.87)	
	Damaged or defective traffic signs	22483(74.83)	7388(24.59)	175(0.58)	
	Road narrowing	15816(69.44)	6528(28.66)	431(1.89)	
	Bumps	1376(80.09)	333(19.38)	9(0.52)	
	Unstable shoulders or nonexistent shoulders	603(63.94)	288(30.54)	52(5.51)	
	Lack of standard road guard	806(58.24)	503(36.34)	75(5.42)	
Roadway defect	Partial road collapse	140(69.65)	55(27.36)	6(2.99)	<0.001
	Defective pavements	277(73.67)	88(23.40)	11(2.93)	
	Acute angle	474(68.20)	187(26.91)	34(4.89)	
	Non-standard grading	264(73.33)	86(23.89)	10(2.78)	
	Defective lighting	571(58.99)	334(34.50)	63(6.51)	
	Slippery road	746(79.87)	169(18.09)	19(2.03)	
	Unknown	219702 (80.29)	53221 (19.450)	700 (0.26)	
	Straight and flat	265713 (76.76)	77884 (22.50)	2570 (0.74)	
	Straight and Uphill/downhill road	10446 (81.44)	2225(17.35)	156(1.22)	
Roadway geometrics	Winding Road and flat	11994 (74.95)	3750(23.43)	258(1.61)	<0.001
	Winding Road and Uphill/downhill	3770 (77.54)	942(19.37)	150(3.09)	
	Unknown	135719 (83.26)	27031 (16.58)	254 (0.16)	
	Dry	382648 (77.65)	106963 (21.71)	3146 (0.64)	
	Wet	22243 (85.59)	3545 (13.64)	201(0.77)	
	Freezing and snow	1146 (89.53)	122 (9.53)	12(0.94)	
Roadway surface	Sandy	964 (86.23)	140 (12.52)	14(1.25)	<0.001
	Muddy	69 (81.18)	15 (17.65)	1(1.18)	
	Oily	22 (75.86)	6 (20.69)	1(3.45)	
	Unknown	20550 (95.12)	1041 (4.82)	13(.06)	

A total of 393,310 (72.45%) RTCs occurred at daytime, followed by 122,957, (22.65%) at night time. As observed in Table 2, the proportion of RTCs which led to injury (24.44% at sunrise and 27.16% at sunset) and death (5.43% at sunrise and 1.43% at sunset) were significantly higher than those occurring during daytime (20.50% injury and 0.55% death, P < 0.001). Table 4 showed that the odds of injury and death in scene were higher at night, sun rise and sun set than daytime. In this respect, crash was more dangerous at sunrise. 

**Table 4:** Association of the type of crash with respect to a number of environments and road related factors by multinomial logistic regression.

		Injury vs. Non-injured vehicular crash		Death in scene vs. Non-injured vehicular crash	
		OR (95% C.I.)**	P value	OR (95% C.I.)**	P value
	Day	1	-	1	-
Scene light	Night	1.22 (1.20-1.24)	<0.001	1.70 (1.58-1.83)	<0.001
	Sun rise	1.34 (1.18-1.52)	<0.001	11.20 (8.72-14.38)	<0.001
	Sun set	1.47 (1.38-1.55)	<0.001	2.90 (2.32-3.62)	<0.001
	Clear	1	-	1	-
	Foggy	0.79 (0.69-0.90)	0.001	1.92 (1.22-30.3)	<0.001
	Snowy	0.37 (0.31-0.43)	<0.001	1.25 (0.76-2.04)	<0.001
Weather	Rainy	0.53 (0.51-0.55)	<0.001	0.95 (0.80-1.12)	0.265
	Storm	1.11 (0.76-1.63)	0.583	2.22 (0.55-8.98)	0.509
	Cloudy	0.81 (0.78-0.84)	<0.001	1.3 (1.13-1.49)	0.382
	Dust	1.45 (1.07-1.96)	0.015	9.27 (5.02-17.13)	0.005
	Motorway	1	-	1	-
	Road shoulder	1.22 (1.14-1.32)	<0.001	5.05 (4.23-6.04)	<0.001
Place of crash	The middle lane	0.78 (0.70-0.87)	<0.001	2.39 (1.73-3.28)	<0.001
	Roadside	1.9 5 (1.83-2.07)	<0.001	8.7 (7.58-9.97)	<0.001
	Outside of road limit	1.17 (1.02-1.34)	0.022	5.57 (4.10-7.56)	<0.001
	No defect	1	-	1	-
	Damaged or defective traffic signs	1.25 (1.22-1.29)	<0.001	0.71 (0.61-0.83)	<0.001
	Road narrowing	1.57 (1.53-1.62)	<0.001	2.47 (2.22-2.75)	<0.001
	Bumps	0.92 (0.82-1.04)	0.192	0.59 (0.31-1.15)	0.120
	Unstable shoulders or nonexistent shoulders	1.82 (1.58-2.10)	<0.001	7.83 (5.87-10.43)	<0.001
	Lack of standard road guard	2.38 (2.13-2.66)	<0.001	8.45 (6.64-10.75)	<0.001
Roadway defect	Partial road collapse	1.50 (1.10-2.05)	0.011	3.89 (1.72-8.82)	0.001
	Defective pavements	1.21 (0.95-1.54)	0.117	3.61 (1.97-6.60)	<0.001
	Acute angle	1.51 (1.27-1.78)	<0.001	6.51 (4.58-9.25)	<0.001
	Non-standard grading	1.24 (0.97-1.59)	0.080	3.44 (1.83-6.48)	<0.001
	Defective lighting	2.23 (1.95-2.56)	<0.001	10.02 (7.69-13.05)	<0.001
	Slippery road	0.86 (0.73-1.02)	0.087	2.31 (1.46-3.65)	<0.001
	Straight and flat	1	-	1	-
Roadway geometrics	Straight and Uphill/downhill road	0.73 (0.69-0.76)	<0.001	1.54 (1.31-1.82)	<0.001
	Winding Road and flat	1.07 (1.03-1.11)	0.001	2.22 (1.95-2.53)	<0.001
	Winding Road and Uphill/downhill	0.85 (0.79-0.92)	<0.001	4.11 (3.48-4.86)	<0.001
	Dry	1	-	1	-
	Wet	0.91 (0.79-1.05)	0.196	0.52 (0.45-0.60)	<0.001
Roadway surface	Freezing and snow	0.79 (0.44-1.39)	0.405	0.3 (0.17-0.54)	<0.001
	Sandy	0.57 (0.33-0.96)	0.035	0.29 (0.17-0.51)	<0.001
	Muddy or Oily	0.37 (0.09-1.52)	0.169	0.31 (0.07-1.32)	0.112

**OR: odds ratio; 95% C.I.: 95% confidence interval for odds ratio.

As shown in Table 1, the greatest number of crashes was 471,759 (86.90%) which took place in clear weather and only 0.04% of RTCs occurred in dusty weather, which had the highest rate of death compared to other weather conditions (5.07%,P <0.001). The odds of injury was higher in dusty weather and lower in foggy, snowy, rainy and cloudy weather than the clear weather. The odds of death in scene was higher in dusty, foggy, snowy, rainy and stormy weather than in clear weather. Crash was more dangerous in dusty weather (Table 2).

In terms of road surface, 492,757 (90.77%) crashes occurred on dry surfaces, while oily surfaces caused the highest rate of mortality (3.45%, P<0.001).

In regard to RTC- related defects, defective traffic signs (30,046, 5.58%) and road narrowing (22,775, 4.23%) were the most frequently identified defects (Table 1).

Flat straight and winding uphill/downhill road were two roadway geometrics representing the highest and lowest proportions of crashes (346,168, 63.77% and 4,862, 0.90%, respectively). In contrast, these two roadway geometrics inflicted the minimum (0.74 %) and maximum (3.09%) RTC- related deaths P<0.001).

## Discussion

To our knowledge, similar studies on the association between environmental factors and RTCs have not been reported from Iran. Our study showed that most crashes occurred during daytime hours, which were considerably higher than those observed at sunrise or sunset. The working day traffic accounts for greater number of crashes. In an earlier study in Iran, the number of RTCs was reported to depend on the time of the day,^15^ where higher incidence of car crashes was found during rush hour.

We found that the proportion of RTCs which led to injury and death was significantly higher at sunrise or sunset than daytime hours. The highest rates of RTC fatalities at sunrise could be due to poor visibility because of insufficient light at sunrise and driver's sleepiness, inattention and lack of alertness at sunrise which resulted from sleep deprivation during the night. Previous studies reported the higher risk of death or severe injury due to RTC, which was significantly related to driving in poor visibility and also driving at night time. ^16-18^

Not surprisingly our study showed that traffic crashes were most prevalent on clear weather conditions. This could be the result of climate of Iran where the weather is usually clear and sunny in most days. Iran's climate ranges from arid or semiarid in most parts and the mean annual rainfall is reported 332 millimeters.^19,20^ Clear weather had the lowest death rate among all weather conditions. In this context, dusty weather had the highest death rate compared to other weather conditions. According to the study of Shankar et al., the maximum rainfall played a significant role in road traffic crashes.^9^ The higher rates of RTC fatalities in non-clear weather could be explained by (a) poor visibility due to rainy, snowy or sandy weather (b) the road surface which may be more slippery in non-clear weather, thus reducing the vehicle-roadway friction. Previous study by Hijar et al., in Mexico showed a definite association of adverse environmental conditions such as rain, fog, and wet pavement as well as driving in daylight with traffic crash. ^21^ Kashani et al., in their recent study revealed that weather and road surface conditions, shoulder type and road width, lighting as well as location type are less important variables, influencing the injury severity by traffic crashes than the use of seat belt, cause of crash and collision type. ^22^

In our study, winding uphill/downhill road was the roadway geometry with the highest rate of RTC- related death. This roadway geometry limits the driver's vision and causes difficult control of vehicle at crash time with subsequent increase in fatal RTC risk. 

For RTCs associated with known roadway defect, we found defective traffic signs and road narrowing to be the most frequent defects. Traffic signals and signs are universally accepted interventions which are effective in reducing RTC- related injuries and deaths.^23^ In a recent study conducted on the main factors affecting traffic crashes in Iran, it was observed that most rural accidents occurred in the main roads and highways. The main accident-related factors were wider roads, denser traffic, road narrowing and ignoring traffic regulations. The crashes occurring in highway resulted from the lack of appropriate road repair and installing suitable traffic signs.^24^

In conclusion, in our analysis of traffic crash in Iran, we found light in the scene of crash, weather condition, roadway geometrics and road surface to be the important contributors to traffic crash injuries and deaths. Based on the results obtained, it is recommended to introduce changes to the road-associated environmental conditions, a measure highly beneficial to reducing RTC-related injuries and deaths.

**Limitations of the study**

The limitations of our study were mainly due to the variables available in the Traffic Police Department data bank. Police mortality records comprised only about 45% of total scene deaths. This under-registration was firstly due to the fact that a large proportion of crash victims, receiving severe and life-threatening injuries, were immediately transferred to the hospitals before inspection by attending traffic police. Therefore, these cases were not reflected in the final death report of traffic police data bank. Also police officers did not record traffic crashes under confusing circumstances.

Secondly, there were missing data for each variable. The police probably overlooked a number of data due to underreporting of less severe injuries and collisions.

The third limitation was that police crash database only accounted for in-scene deaths while the real number of fatalities were higher than those reported by the police. According to the study of Ayati et al.^2^ the number of deaths reported by the police should be multiplied by 2.5 to cover those who died before or after reaching the hospitals. 

The fourth limitation of this study was that it focused on the environmental factors rather than on associated main risk factors. Furthermore, after extensive literature review, we did not find any study that analyzed the association between RTCs and multiple environmental factors including weather conditions, place of crash, roadway defect, roadway geometrics and roadway surface, as completely as our investigation. 
